# Approaching closed spherical, full-view detection for photoacoustic tomography

**DOI:** 10.1117/1.JBO.27.8.086004

**Published:** 2022-08-30

**Authors:** Lawrence C. M. Yip, Parsa Omidi, Elina Rascevska, Jeffrey J. L. Carson

**Affiliations:** a Lawson Health Research Institute, Imaging Program, London, Ontario, Canada; b Western University, Schulich School of Medicine and Dentistry, Department of Medical Biophysics, London, Ontario, Canada; c Western University, School of Biomedical Engineering, London, Ontario, Canada; d Western University, Schulich School of Medicine and Dentistry, Department of Surgery, London, Ontario, Canada

**Keywords:** photoacoustic tomography, full-view, ultrasound, limited-view, artifacts

## Abstract

**Significance:**

Photoacoustic tomography (PAT) is a widely explored imaging modality and has excellent potential for clinical applications. On the acoustic detection side, limited-view angle and limited-bandwidth are common key issues in PAT systems that result in unwanted artifacts. While analytical and simulation studies of limited-view artifacts are fairly extensive, experimental setups capable of comparing limited-view to an ideal full-view case are lacking.

**Aims:**

A custom ring-shaped detector array was assembled and mounted to a 6-axis robot, then rotated and translated to achieve up to 3.8π steradian view angle coverage of an imaged object.

**Approach:**

Minimization of negativity artifacts and phantom imaging were used to optimize the system, followed by demonstrative imaging of a star contrast phantom, a synthetic breast tumor specimen phantom, and a vascular phantom.

**Results:**

Optimization of the angular/rotation scans found $\approx 2^{12}$ effective detectors were needed for high-quality images, while 15-mm steps were used to increase the field of view as required depending on the size of the imaged object. Example phantoms were clearly imaged with all discerning features visible and minimal artifacts.

**Conclusions:**

A near full-view closed spherical system has been developed, paving the way for future work demonstrating experimentally the significant advantages of using a full-view PAT setup.

## 1 Introduction

Photoacoustic tomography (PAT) is a three-dimensional (3D) imaging modality that uses a combination of diffuse laser light and ultrasonic detectors to provide soft tissue contrast comparable to MRI, in a format and cost comparable to ultrasound, all without using ionizing radiation. Over the last two decades, PAT has been proposed for a multitude of medical and animal use cases, including for breast cancer screening,^1^^,^^2^ prostate treatment guidance,^3^^,^^4^ tumor margin assessment,^5^^,^^6^ and small limb imaging.^7^ Unfortunately, while recent technological and application developments have been impressive, PAT has yet to reach its full potential, in large part due to the difficulty in obtaining complete data coverage.

In PAT, it can be broadly said that the contrast mechanism is provided by absorbed laser light, while depth information and resolution are determined by characteristics of ultrasonic waves^8^ and a system’s ability to accurately and fully detect them. Photoacoustic signals generated by an optical absorber propagate outward in all directions. In addition, for certain geometries of objects (e.g., cylindrical blood vessels), the main photoacoustic signal will propagate anisotropically.^9^ All of these signals produced by an object must be recorded at a detection surface to be accurately imaged. For an ideal 3D system, an infinitely large number of discrete detectors or a continuous detection surface spread over the entirety (4π steradians) of a sphere would provide the most accurate image reconstruction.^10^ However, this is not possible in practice and there must be some degree of sparsity in the detection array. Except for a few cases involving integrating^11^ or optical detectors,^12^ detection elements in PAT arrays are typically discrete, physical objects with finite diameters that cannot overlap.

Early systems used single-element transducers^13^ or linear-array ultrasound probes.^14^^,^^15^ In such limited-view angle situations, it would be expected that there would be blurring and loss of high-frequency signals.^10^ To improve the view angle, many groups have used planar,^16^ cylindrical,^7^^,^^17^ and hemispherical arrays^1^^,^^2^^,^^5^ with varying degrees of success. In each case, about 2π steradians is the maximum achievable view angle.^10^ This is problematic as signals traveling outside of the detection area are still lost, and to compound this, reconstruction algorithms are generally designed for full-view and result in significant artifacts in a limited-view scenario.^10^

Other proposed solutions have included the use of acoustic reflectors to multiply the physical detectors,^18^^–^^22^ acoustic backscatterers as virtual detectors,^23^ and integrating line arrays.^24^ Furthermore, substantial research has been conducted to improve reconstruction methods for limited-view cases. These include analytical solutions,^25^^,^^26^ using sparsity-based beamforming,^27^ applying compressed sensing,^28^^,^^29^ iterative reconstruction methods,^30^^,^^31^ and more recently, artificial intelligence related methods, such as machine learning and deep convolutional neural networks, have been widely investigated to improve reconstruction of images with sparse coverage or limited-view.^32^^–^^35^

Another component of PAT involves the ultrasonic detection elements; where, unlike in traditional ultrasound imaging, photoacoustic signals emitted by an imaged object (absorber) are typically broadband in nature and require a wideband—or ideally infinitely broadband—transducer to properly detect these signals.^36^^,^^37^ Early PAT systems used commercially available transducers intended for ultrasound imaging, which were not ideal as they are typically narrowband in nature and often best detect frequencies in the megahertz regime.^15^ It has also been shown that lower frequency transducers provide key bulk tissue information that is missed by higher frequency transducers.^38^ However, this must be balanced against the opposing problem, where low frequency transducers may be unable to resolve high frequency information, such as from fine capillaries.^36^^,^^37^^,^^39^ For this system, we incorporated wideband, relatively low frequency transducers to find a good balance of spatial resolution and bulk tissue contrast.

With many reconstruction algorithms, including in backprojection-based reconstruction methods—which are some of the most widely used due to their simplicity and computational efficiency—edge artifacts with negative values are commonly seen. Calling them “negativity artifacts,” Shen et al. recently explored the causes and impacts of these artifacts.^40^ Briefly, the impulse response in a backprojection algorithm is only a delta function under ideal circumstances—which is never the case in experiments. Deviations from this ideal, whether due to limited bandwidth or limited view, result in distorted initial pressure images and, therefore, negativity artifacts. Extending this research, which was primarily simulation-based, we demonstrate how quantification of negativity artifacts can be used to optimize parameters in system design.

With decades of research in PAT now available, there is no shortage of research systems with a variety of array shapes having been explored. However, we are unaware of any system available approaching a closed spherical detection geometry, i.e., approaching the ideal 4π steradian view angle. To address this, we present a PAT system designed around a broadband ring array and a highly flexible 6-axis robot. Angular scan parameters were optimized by measuring negativity artifacts, then translation step size was determined through phantom imaging. This resulted in a near-full-view closed spherical PAT system. Using a star phantom, a synthetic breast tumor phantom, and a vascular phantom, image comparison was then performed between the full-view system and emulated limited-view geometries.

## 2 Materials and Methods

### 2.1 PAT System Description

**Fig. 1:**
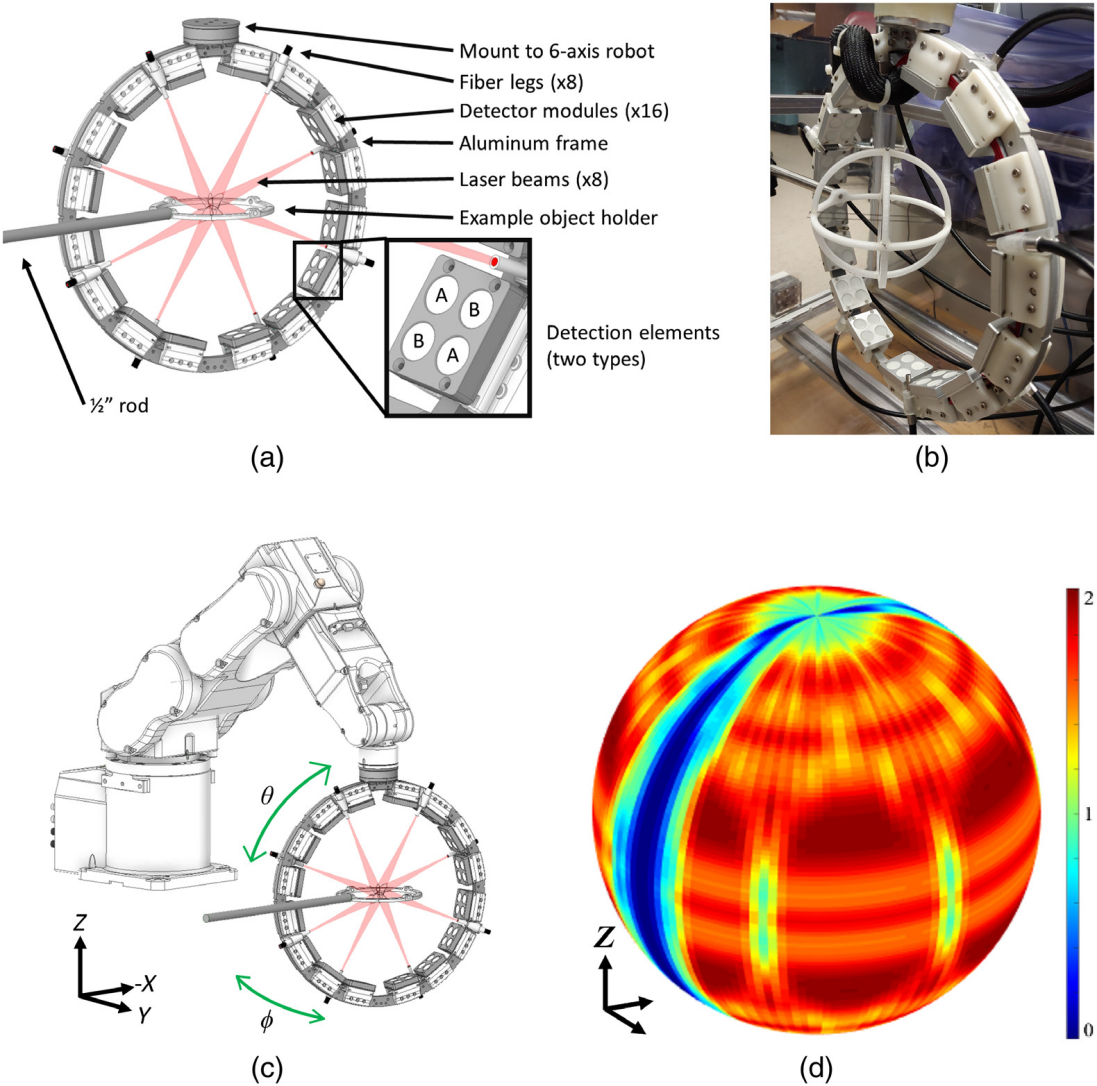
PAT system design illustrating the detection and illumination setups. (a) CAD drawing of the circular ring array with 16 detector modules (two types, A and B) and eight fiber legs surrounding one example of an object holder. (b) Photograph of the ring array. (c) CAD drawing of the array mounted to the 6-axis robot with arrows in green denoting azimuthal angle *phi* ϕ and elevational angle *theta* θ rotational directions. (d) Heatmap showing the relative density of detectors across the sphere of a single 64 position/3584 effective detector scan. Note the cutouts on the sides to allow the 1/2" rod of the object holder to extend into the imaging area. System components not depicted (nonexhaustive): water tank, robot controller, DAQ/ADC system, host controller PC, and illumination laser.

In this work, we assembled a PAT system as shown in Fig. 1. The main components of the system included an illumination laser, an ultrasonic detector array, signal processing hardware (amplification, acquisition, and digitization modules), and a 6-axis robot. The system was controlled and data saved through a standard Microsoft Windows 10 workstation running LabVIEW (2014, NI, Austin, Texas).

#### 2.1.1 Illumination

Illumination was provided by a tunable Nd:YAG laser system (Phocus™, Opotek Inc., Carlsbad, California, 680 to 950 nm, 10-Hz pulse repetition rate, 5-ns pulse width) coupled to a high-power eight-legged fused-end fiber bundle (Excelitas Canada Inc., Mississauga, Ontario, Canada, NA 0.37). Pulse-to-pulse light intensity variation was accounted for using a silicon photodiode (DET10A, Thorlabs Inc., Newton, Massachusetts) placed behind one of the mirrors in the light path. The eight output legs were spread out around the frame of the array [Fig. 1(a)] and oriented toward the center of the array at a distance such that the beam width approximately matched the aperture of the acoustic detectors. Unless otherwise stated, all imaging presented herein was performed with 690-nm laser light. To synchronize the system, the external trigger of the laser Q-switch was used to trigger the data acquisition systems and the 6-axis robot.

#### 2.1.2 Acoustic detection and reconstruction

We used technology developed in-house to assemble a total of 64 polyvinylidene fluoride (PVDF)-based unfocused broadband detectors divided into 16 modules (Superior Assemblies, Mississauga, Ontario, Canada). Each module had two lower frequency and two higher frequency elements, with circular apertures of 12.7 mm [Fig. 1(a)].

The modules were mounted to a CNC-machined aluminum circular ring, such that detection surfaces were positioned around an ≈280-mm diameter circle. A total of 16 detector modules were positioned on the ring. Eight were evenly spaced on each side (21.5 deg between modules on the same side and 7.4 deg between adjacent detectors on the same module), with larger gaps at the top and bottom to decrease oversampling near the poles [Fig. 1(a)]. The ring was in turn mounted to a 6-axis robot [Fig. 1(c)] (C3, Epson America Inc., Los Alamitos, California) and submerged in a polycarbonate water tank. Each module was internally preamplified then connected to a modular custom analog-to-digital converter and data acquisition system (Multimagnetics Inc., London, Ontario, Canada, 14-bit dynamic range, 50-MHz sampling rate), which then transferred the data to the host PC through several USB 2.0 interfaces. Unless otherwise stated, data were fluence-corrected, and image reconstruction was performed using a delay and sum algorithm.^41^

The 6-axis robot enabled translation (in Cartesian X, Y, and Z) and rotation [in spherical phi (ϕ) and theta (θ) angles] of the array. This effectively allowed the circular array to approach a closed spherical geometry, with the exception of a narrow gap to allow clearance for a support rod for the object [Fig. 1(d)].

### 2.2 System Optimization and Characterization

#### 2.2.1 Frequency and spatial resolution characterization

Frequency characterization methodology was adapted from Ref. 42. Briefly, 2% w/v agarose (VWRVN605, CAS #9012-36-6, VWR International, Radnor, Pennsylvania) was added to pure India ink (Speedball 3378 Super Black India Ink, Speedball Art, Statesville, North Carolina) to form a flat slab, which was placed parallel to each detector with both submerged underwater. For a subset of 8 out of the 64 detectors, an impulse signal was then acquired, and the frequency content was analyzed using the fast Fourier transform.

Spatial resolution was then determined using images acquired with a single angular scan of 64 positions/3584 effective detectors. Two crossed nylon threads (Coats black upholstery thread, Coats Group PLC, Uxbridge, United Kingdom), each with a nominal diameter of 0.400 mm, were used as the resolution target. A maximum intensity projection (MIP) was then taken of the image, and the full-width at half-maximum (FWHM) was used to determine the effective resolution.

#### 2.2.2 Angular scan optimization

When using a spherical detection geometry, the number of detectors spread over the detection surface influences the intensity of negativity artifacts in a reconstructed image.^40^ To determine the ideal number and size of rotation steps needed, the detector array was rotated in such a way to approximate a spectrum of evenly distributed detectors, ranging from 196 to 131,880 effective detectors.

A 9-mm 2% w/v agarose sphere with 0.02% v/v India ink and 2% v/v Intralipid (Fresenius Kabi, Toronto, Ontario, Canada) was held in place using a 2-mm hollow glass pipette and imaged. Analysis was performed on the 3D image as well as on each cross-sectional plane (XY, XZ, and YZ) intersecting the middle of the sphere. In each case, image values (2D or 3D) were scaled such that positive values fit between 0 and 1. Resulting maximum negative intensity values were then graphed against the number of detectors to determine the optimal number/density of scan positions (more scan positions = more time to acquire an image).

The advantages of using a full-view spherical system were discussed in Sec. 1. To experimentally demonstrate this advantage, negativity analysis was also performed on images reconstructed with data acquired using subsets of the ring array. These included using the top half of the array (hemispherical), using only the top 16 detection elements, and using only the top eight detection elements. In each case, the same detector density was maintained. An additional data point was also included using double the detector density (same total number of detectors as the spherical case) with the hemispherical geometry.

#### 2.2.3 Field of view determination

The large aperture PVDF detection elements used provided excellent sensitivity, but being unfocused, were also extremely directional leading to a rather small field of view (FoV). The highly directional detection elements led to loss of spatial resolution and therefore distortions at the edges of the FoV.^43^^,^^44^ To image larger objects with this system, the angular scan optimized in Sec. 2.2.2 had to be repeated at specific intervals to fully image a desired object.

To quantify the FoV, we imaged a star phantom made of 2% w/v agarose and 2% v/v Intralipid to increase optical scattering. Absorbing sections also contained 0.015% v/v India ink. A two-piece mold was 3D printed, with the upper section printed out of resin (Clear Resin, Form 2, Formlabs, Somerville, Massachusetts) and the lower portion out of clear polyethylene terephthalate glycol (PETG, HDglass, FormFutura, Nijmegen, The Netherlands). Stereolithography for the upper portion was chosen for its better printing accuracy, while fused deposition modeling and clear PETG were chosen for the lower section as it also functioned as a support frame for the phantom. In our testing, clear PETG absorbed the least near-infrared light (and therefore emitted the least photoacoustic signal) compared to other readily available filaments or resin-based 3D printing materials. The phantom consisted of alternating absorbing and nonabsorbing sections. In each section, a 10-mm-diameter sphere was also embedded. Absorbing sections had a nonabsorbing sphere, and vice versa.

A single angular scan was performed with the star phantom approximately centered and laying horizontally in the XY plane, then repeated with the phantom rotated to lie vertically in the XZ plane. From these images, line profiles in each direction were taken to determine the FWHM. For subsequent imaging, a value slightly smaller than the FWHM was chosen as the step size.

### 2.3 System Demonstration and Comparison to Limited-View Angle

Having previously identified what the ideal angular scan parameters (Sec. 2.2.2) and translational scan parameters (Sec. 2.2.3) were for scanning a given target, we then proceeded to image three synthetic objects to demonstrate the system’s capabilities. For comparison, the images were reconstructed using the full dataset (spherical) as well as with partial datasets to demonstrate the limited-view effect.

#### 2.3.1 Star phantom

We used the same agarose phantom described in Sec. 2.2.3 but imaged it in its entirety, with 15-mm steps in X and Y. At each translational step, the ϕ angle range was automatically adjusted to maximize the view angle while still avoiding the object holder [Figs. 1(c) and 1(d)].

In addition to qualitative comparison, signal-to-noise ratio (SNR) values were calculated between adjacent sections of the image where high and low intensity signals were side by side. Here, SNR was defined as

$$\frac{{\text{Signal}}_{\mathrm{RMS}}}{{\text{Background}}_{\mathrm{RMS}}},$$ (1)

where RMS is the root mean square of the values in the area in question.

#### 2.3.2 Synthetic *ex vivo* breast tumor phantom

**Fig. 2:**
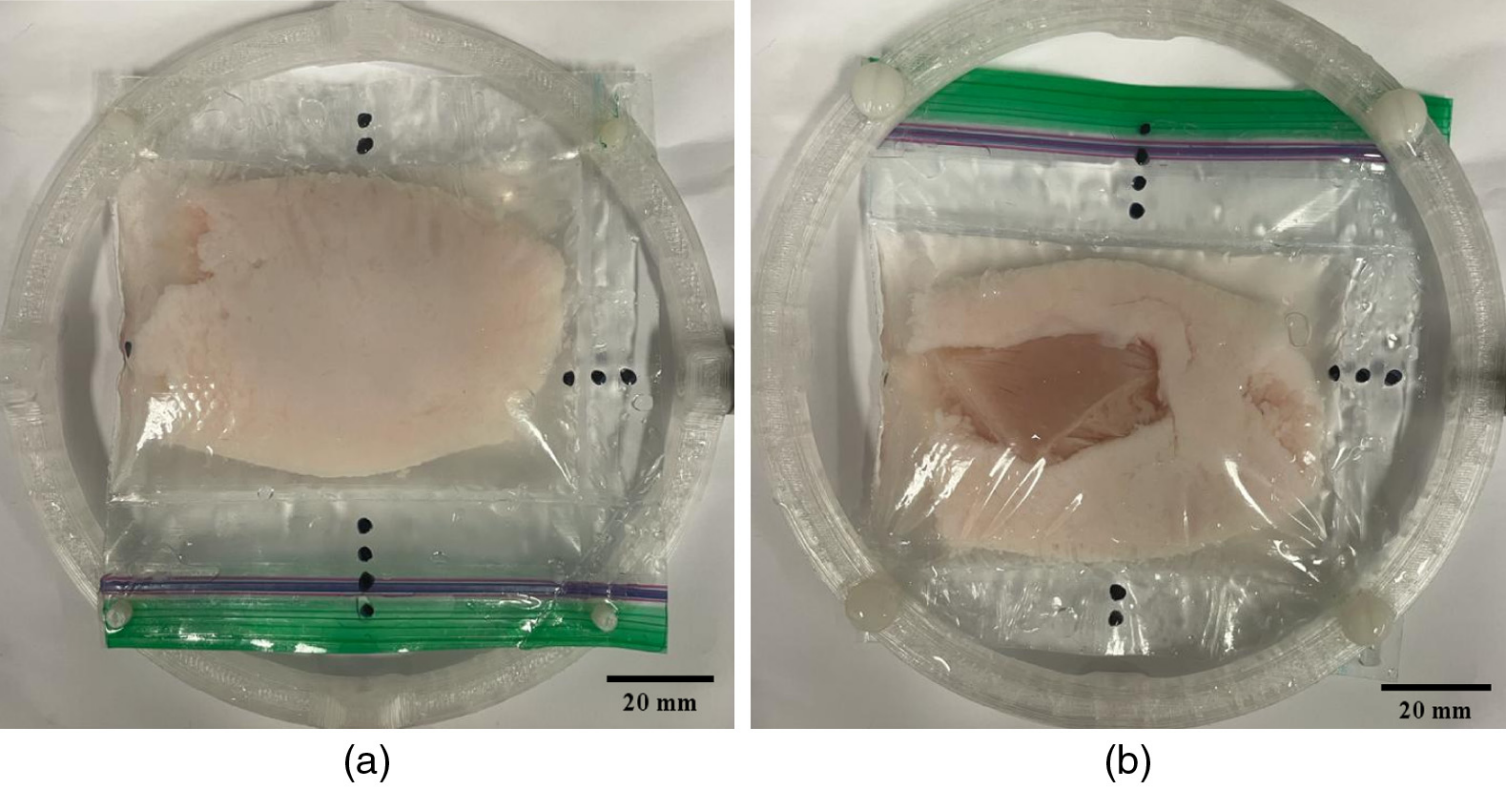
Tumor phantom made of chicken breast (core) and pork belly (outside). Photos of the (a) top and (b) bottom of the phantom mounted to the specimen holder.

One previously explored application of PAT was its capability to image and accurately identify positive versus negative tumor margins in *ex vivo* breast tumor specimens using a lipid-weighted contrast mechanism.^38^ In this paper, we created a synthetic phantom imitating a tumor specimen with a positive margin. A chicken breast core (representing tumor tissue with low lipid content) was wrapped in a thin layer of pork belly fat (representing fatty healthy breast tissue). A gap in the fat layer allowed “tumorous” chicken breast to be exposed, representative of a positive tumor margin. Both the chicken and pork were purchased from a local grocer. Figure 2 shows the construction, with the fat (i.e., high lipid content) wrapped fully around the chicken breast except for one ≈30  mm×20  mm gap at the bottom. This phantom was placed with water in a modified polyethylene Ziploc sandwich bag (S. C. Johnson & Son, Inc., Racine, Wisconsin), then stretched over a 3D-printed frame (PETG with nylon studs). Scan parameters similar to those for the star phantom were used, and an illumination wavelength of 930 nm was used to target lipid contrast.

#### 2.3.3 Vascular phantom

Another popular application of PAT is for the imaging of vascular structures, which may have a tortuous geometry and anisotropic acoustic signal emission. Here, we 3D-printed a vascular phantom using a gray semitranslucent plastic (Grey Pro Resin, Form 2, Formlabs, Somerville, Massachusetts) then embedded it in 2% w/v agarose with 0.5% v/v Intralipid. The phantom was then placed with water in a 2-mil-thick reclosable polyethylene bag (Uline Canada, Milton, Ontario, Canada), then stretched over a 3D-printed frame (PETG with nylon studs). Similar scan parameters were again used, and an illumination wavelength of 800 nm was chosen to maximize laser power. In this particular case, a backprojection reconstruction algorithm with directivity weighting was used.^45^

For this phantom, the SNR for each image was again calculated, with the definition of SNR as follows:

$$\frac{{\text{Signal}}_{\text{mean}}}{{\text{Background}}_{\text{mean}}}.$$ (2)

The CAD model of the phantom was used as a reference to create a binary mask. This was applied to each PAT image, with the area under the mask defined as signal and the remainder as background.

## 3 Results

### 3.1 Frequency and Spatial Resolution Characterization

**Fig. 3:**
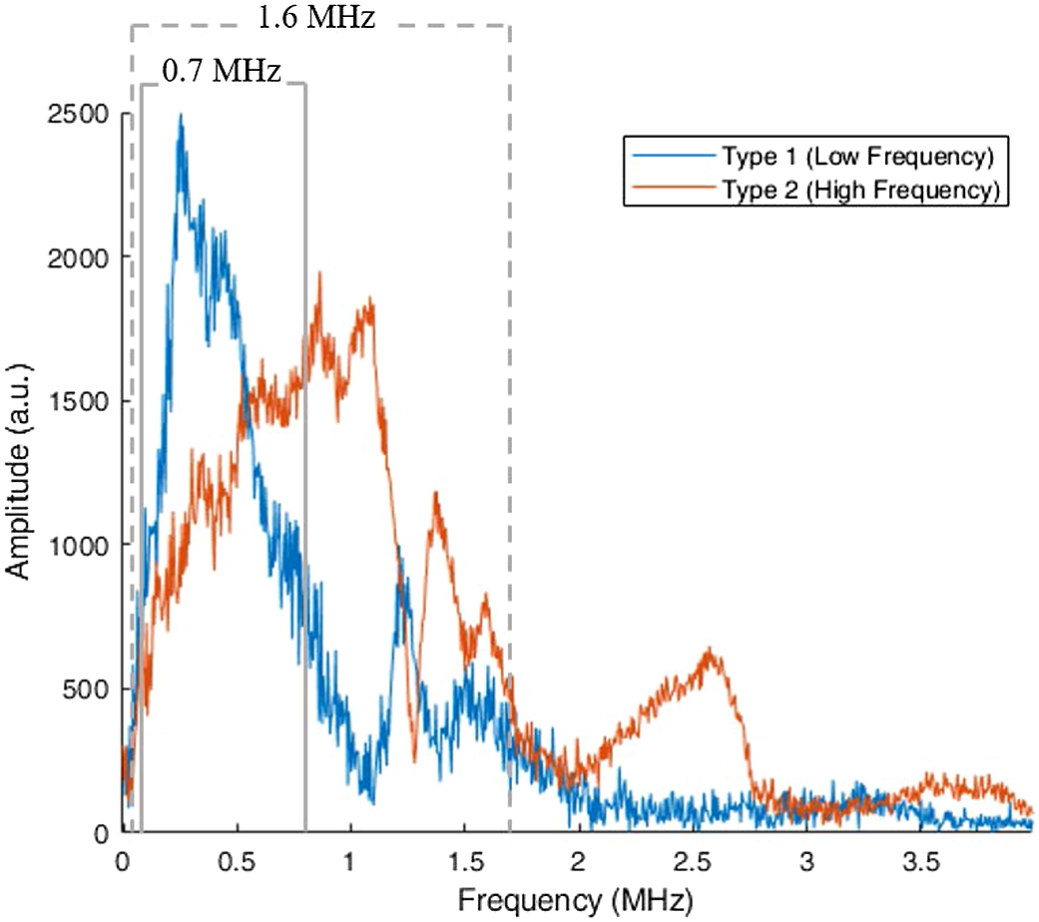
Fourier transform amplitude of sample impulse RF signals showing that the bandwidth (at −6  dB) of the lower frequency elements is 0.7 MHz, and the higher frequency elements, 1.6 MHz. The point source was created by adding 2% w/v agarose to pure India ink, which when placed parallel to the detection elements formed a point source.

An impulse signal was analyzed using the fast Fourier transform, resulting in 0.4-MHz peak frequency (160% one-way bandwidth at −6  dB) and 0.9-MHz peak frequency (185% one-way bandwidth at −6  dB) detectors (Fig. 3).

**Fig. 4:**
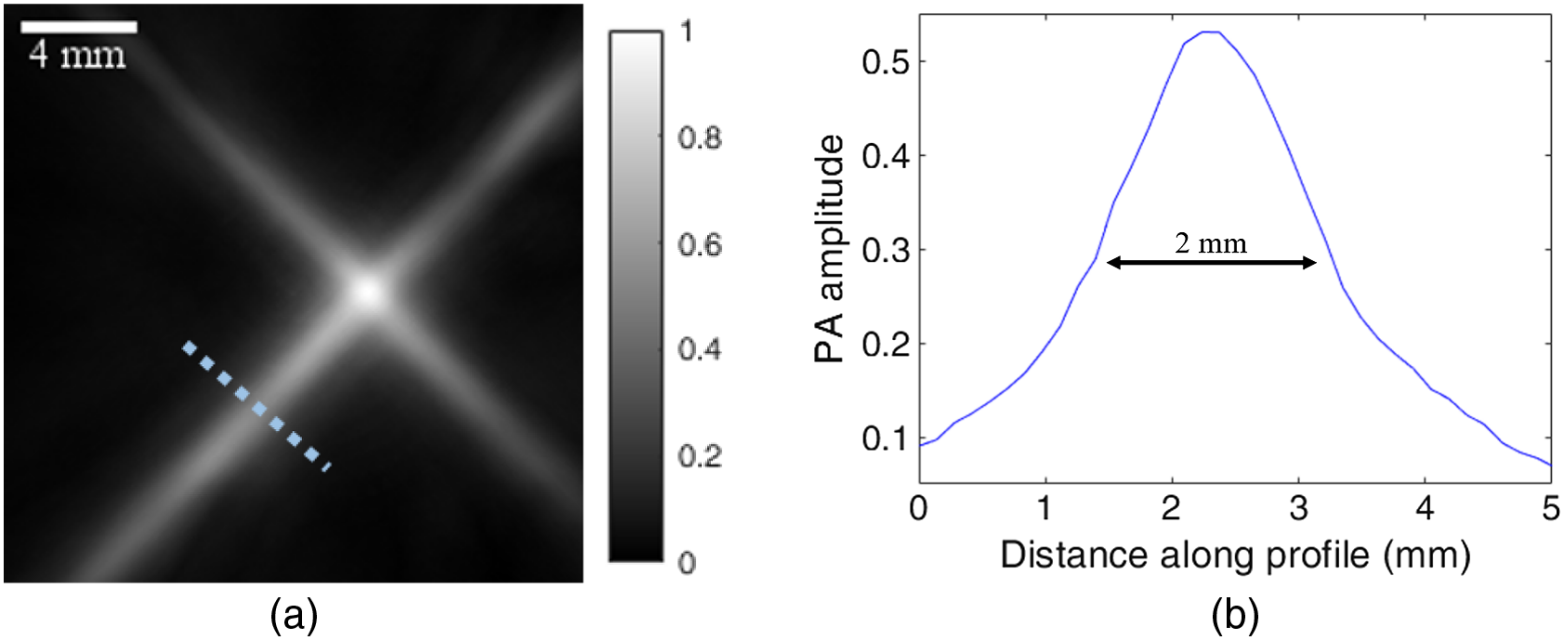
Example system spatial resolution determination. (a) A MIP of two crossed nylon threads (Coats black upholstery thread, Coats & Clark, United Kingdom), each with a nominal diameter of 0.400 mm, using all detectors. (b) Photoacoustic signal amplitude along the dashed line in (a).

Spatial resolution analysis was performed for three cases: only high frequency detectors, only low frequency detectors, and with all the detectors (Fig. 4). The resolution, defined as the FWHM of the amplitudes, was found to be 0.7, 2.0, and 2.0 mm, respectively.

### 3.2 Angular Scan Optimization

As previously mentioned, the 16 detector modules were positioned on a ring with eight evenly spaced on each side [Fig. 1(a)]. Based on the spacing between adjacent modules, a 172.6-deg rotation in ϕ and a 14.1-deg rotation in θ were needed to fully and evenly fill in the 4π steradians required for a full sphere. However, to accommodate the rod holder, the azimuthal angle rotation was reduced to ≈163.5  deg [Fig. 1(d)], resulting in a total spherical view angle equivalent to ≈3.8π steradians. To further reduce oversampling at the top and bottom poles, data from eight (out of 16) detector elements (four of each type) located on the four modules nearest the top and bottom poles were omitted from all analyses.

**Fig. 5:**
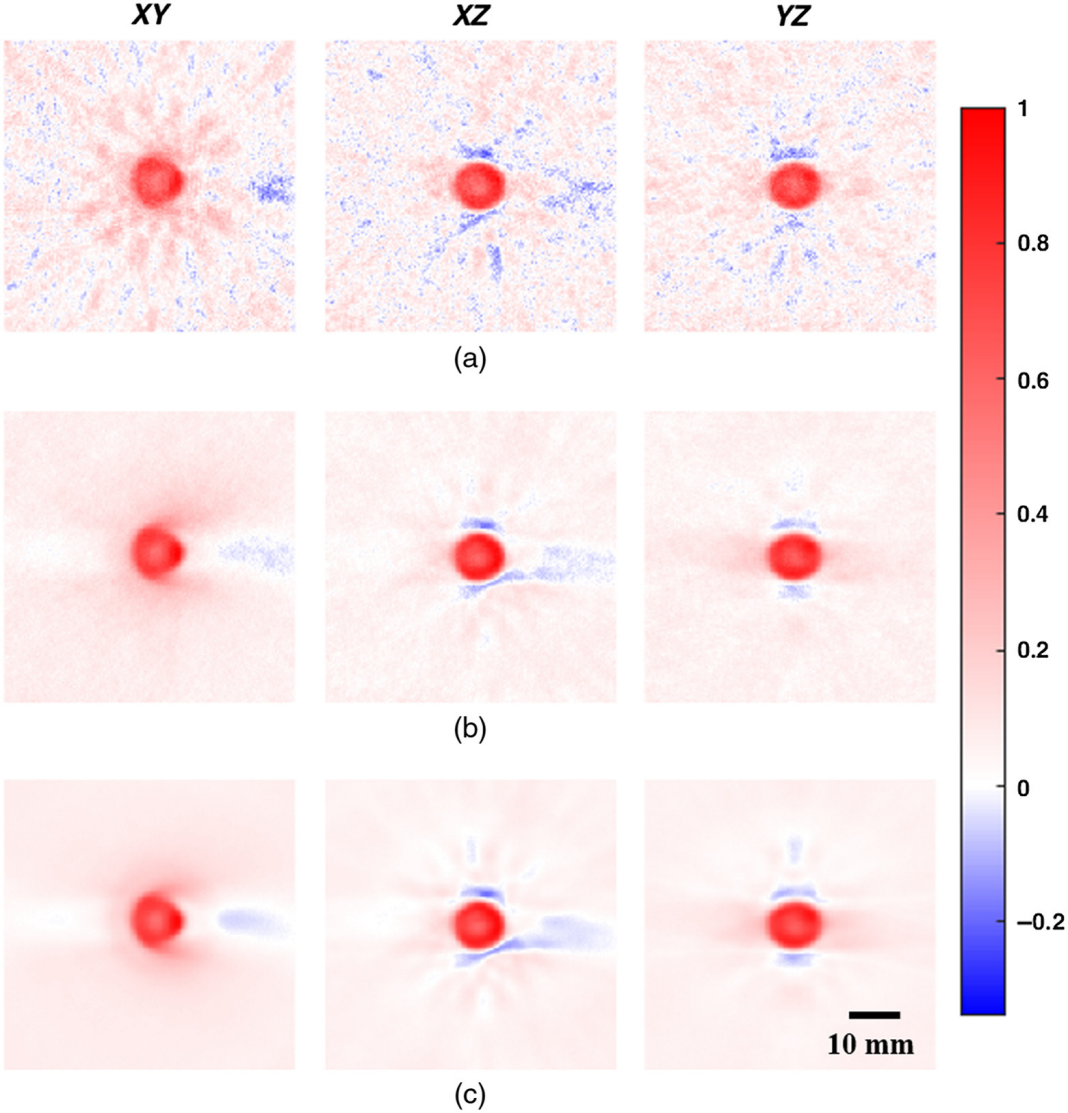
Example cross-sectional slices of a 9-mm agarose sphere containing 2% w/v agarose, 0.02% v/v India ink, and 2% v/v Intralipid reconstructed using (a) 195, (b) 5376, and (c) 131,880 effective detectors spread across a spherical shell. All image intensity values were scaled to fit positive values in each 3D volume between 0 and 1. Intensity bar applies to all images in the figure.

To optimize angular scanning of a single FoV, an absorbing and scattering agarose sphere was scanned at 1-deg increments. Partial datasets were then extracted to represent decreasing numbers of effective detectors, and each dataset was reconstructed and analyzed. Example cross-sectional slices are shown for 195 [Fig. 5(a)], 5376 [Fig. 5(b)], and 131,880 effective detectors [Fig. 5(c)].

At 195 effective detectors [Fig. 5(a)], noticeable speckle-like features and negativity artifacts were present throughout the background of the image. The absorbing sphere was well defined but appeared to have rays of higher intensity signals coming out of it in the XY image. In addition, in the XZ and YZ images, concentrations of negativity artifacts were also present just above and just below the sphere.

With both 5376 [Fig. 5(b)] and 131,880 effective detectors [Fig. 5(c)], the background was very smooth with no visible negativity artifacts. The absorbing sphere was well defined, and the only remaining negativity artifacts were those just above and below the sphere in the XZ and YZ planes and coming in from the right side of the image in XY and XZ planes. In both cases, the strength of the negativity signals decreased as compared to 195 detectors. Between the two, the only visible differences were slightly smoother features, artifacts, and background in the 131,880-detector images.

**Fig. 6:**
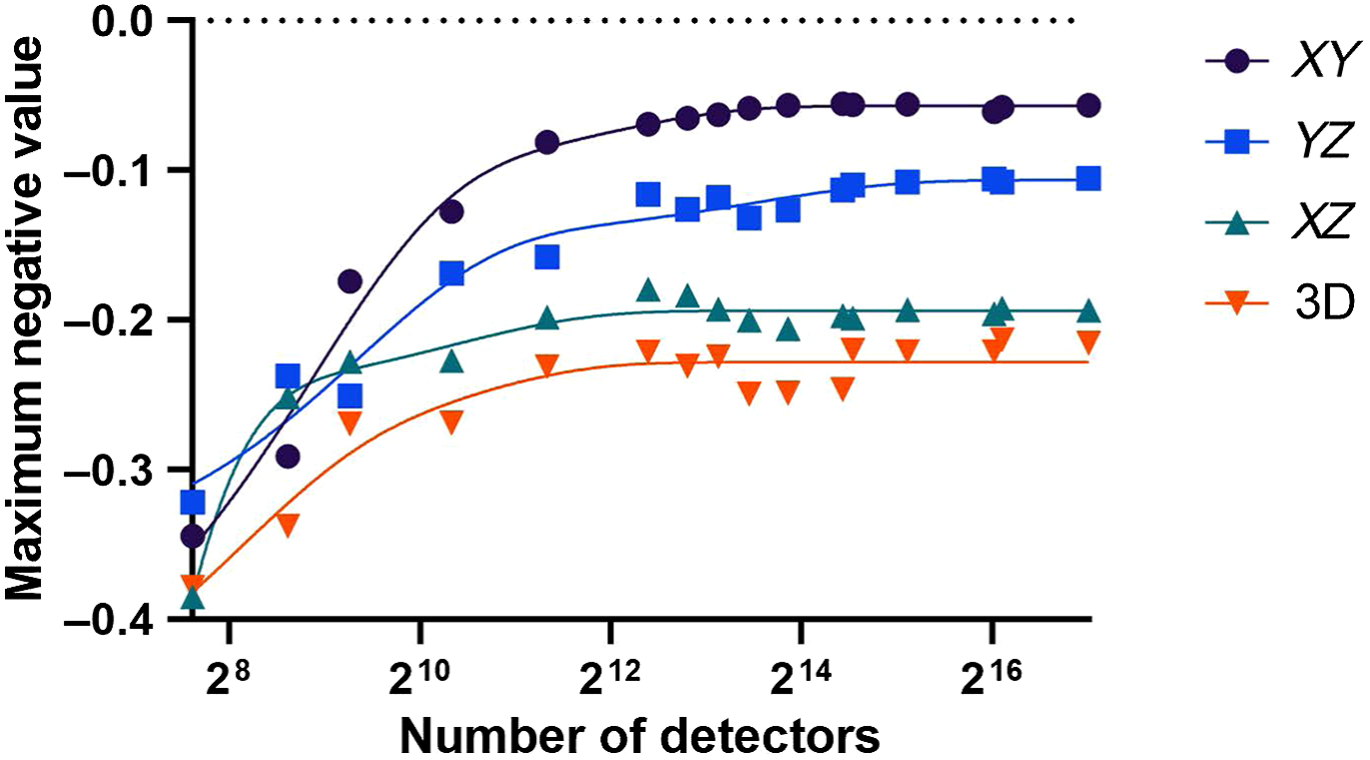
Impact of number of effective detectors spread across a spherical shell (i.e., detector density) on the negativity artifacts. Data series for analysis of the full 3D volume, and each cross-sectional slice are shown. Lines of best fit calculated using a least squares fit to a two phase decay equation, for qualitative comparison purposes only.

Shown in Fig. 6 are the maximum negativity values for the 3D volume as well as for each of the XY, XZ, and YZ cross-sectional slices for each number of effective detectors used. A clear trend can be seen in each data series where the negative values when using relatively few detectors were quite large with values between −0.3 and −0.4, but rapidly decreased with increasing number of detectors, beginning to inflect toward a plateau at ≈4000 effective detectors. The 3D data series plateaued at −0.25, the XZ data series at −0.2, the YZ data series at −0.12, and the XZ data series at −0.06. These values were also visible in the example cross-sectional slices (Fig. 5), where negative values remained present in all the images, including those using all 131,880 detectors [Fig. 5(c)].

**Fig. 7:**
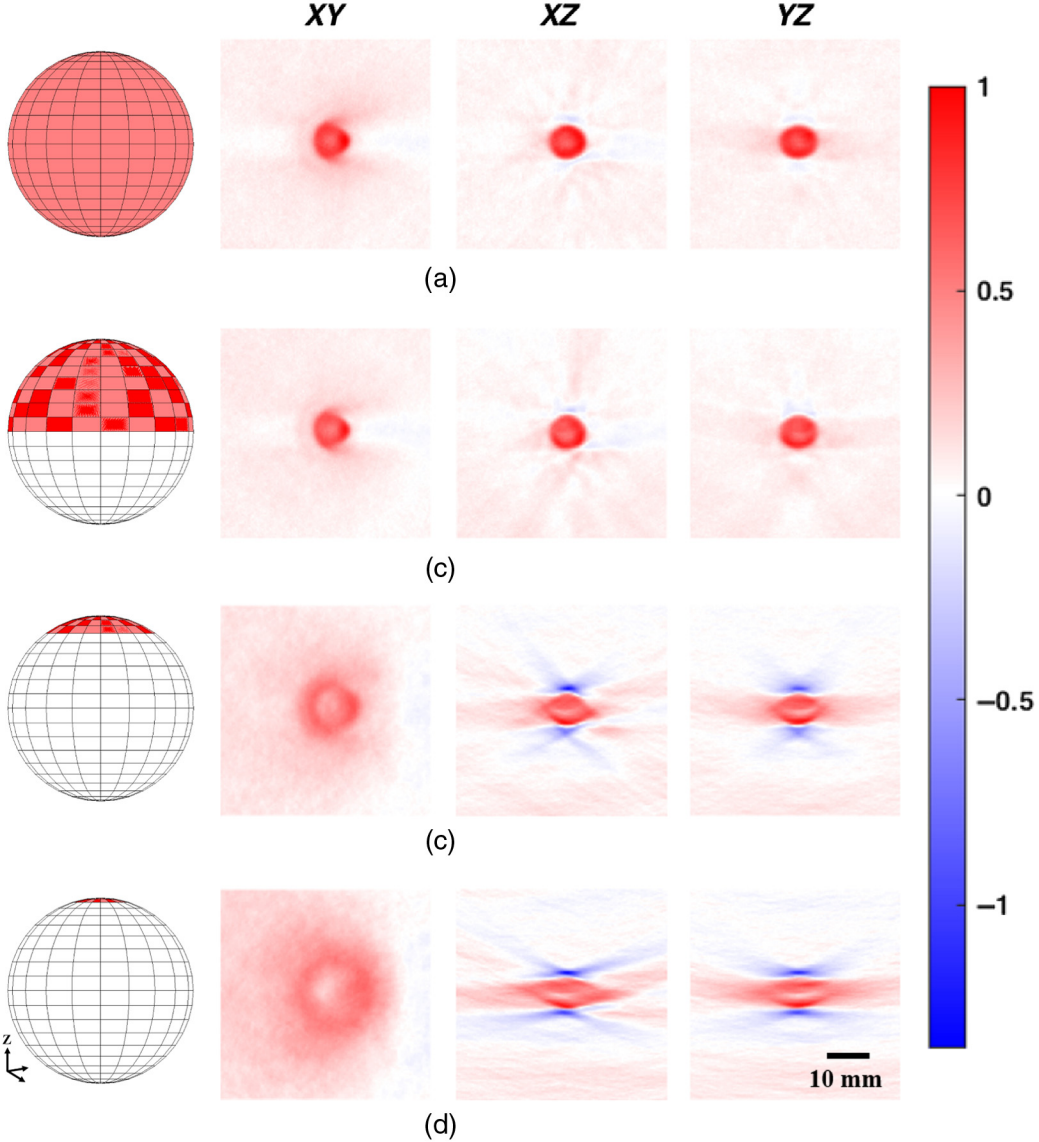
Example cross-sectional slices of a 9-mm agarose sphere containing 2% w/v agarose, 0.02% v/v India ink, and 2% v/v Intralipid acquired using (a) full spherical coverage, (b) hemispherical coverage, (c) top 16 detection elements, and (d) top eight detection elements. All image intensity values were scaled to fit positive values in each 3D volume between 0 and 1. Intensity bar applies to all photoacoustic images in the figure.

**Fig. 8:**
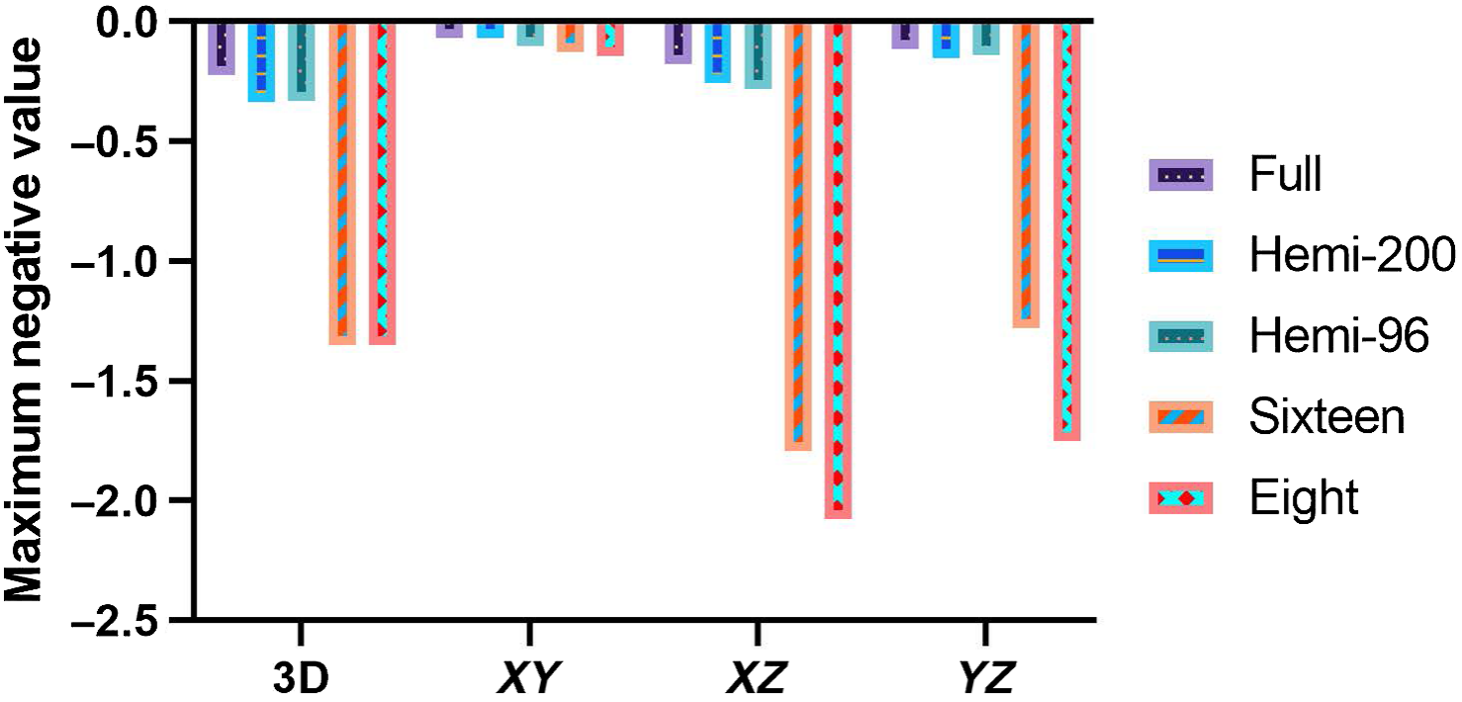
Comparison of maximum negative values between different geometries of detection arrays. Data from the same 96-position dataset were used (i.e., same density of detectors) for the full-view spherical (Full), hemispherical (Hemi-96), top 16 detectors (Sixteen), and top eight detectors (Eight) columns. An additional datapoint using a 200-position dataset was also included (Hemi-200).

At the 5376 detector density, additional image reconstructions were investigated using only the top half of the ring array (effectively a hemispherical array), only the top 16 detection elements, and only the top eight detection elements (Fig. 7). Visually, the full [Fig. 7(a)] and hemispherical [Fig. 7(b)] results were very similar, while the 16 [Fig. 7(c)] and 8 [Fig. 7(d)] element results showed an ill-defined sphere and strong smearing in the X and Y directions. Negativity artifacts were also of a much higher amplitude. Compared to a full spherical array, the negativity values found were overall larger, especially with the 16 and 8 element data (Fig. 8). Values for a hemispherical reconstruction using about the same number of effective detectors as for the full sphere were also included. Hemispherical results for both cases appeared very similar, and only slightly worse than the full sphere results.

Using the partial dataset nearest to the optimal number of detectors (3584), a heatmap [Fig. 1(d)] was generated to show the relative distribution of effective detectors across the sphere. The narrow opening along each side lacking detectors can be seen, with the detectors relatively evenly spread across the rest of the surface, each area having either one detector (green) or two detectors (red).

### 3.3 Field of View Determination

**Fig. 9:**
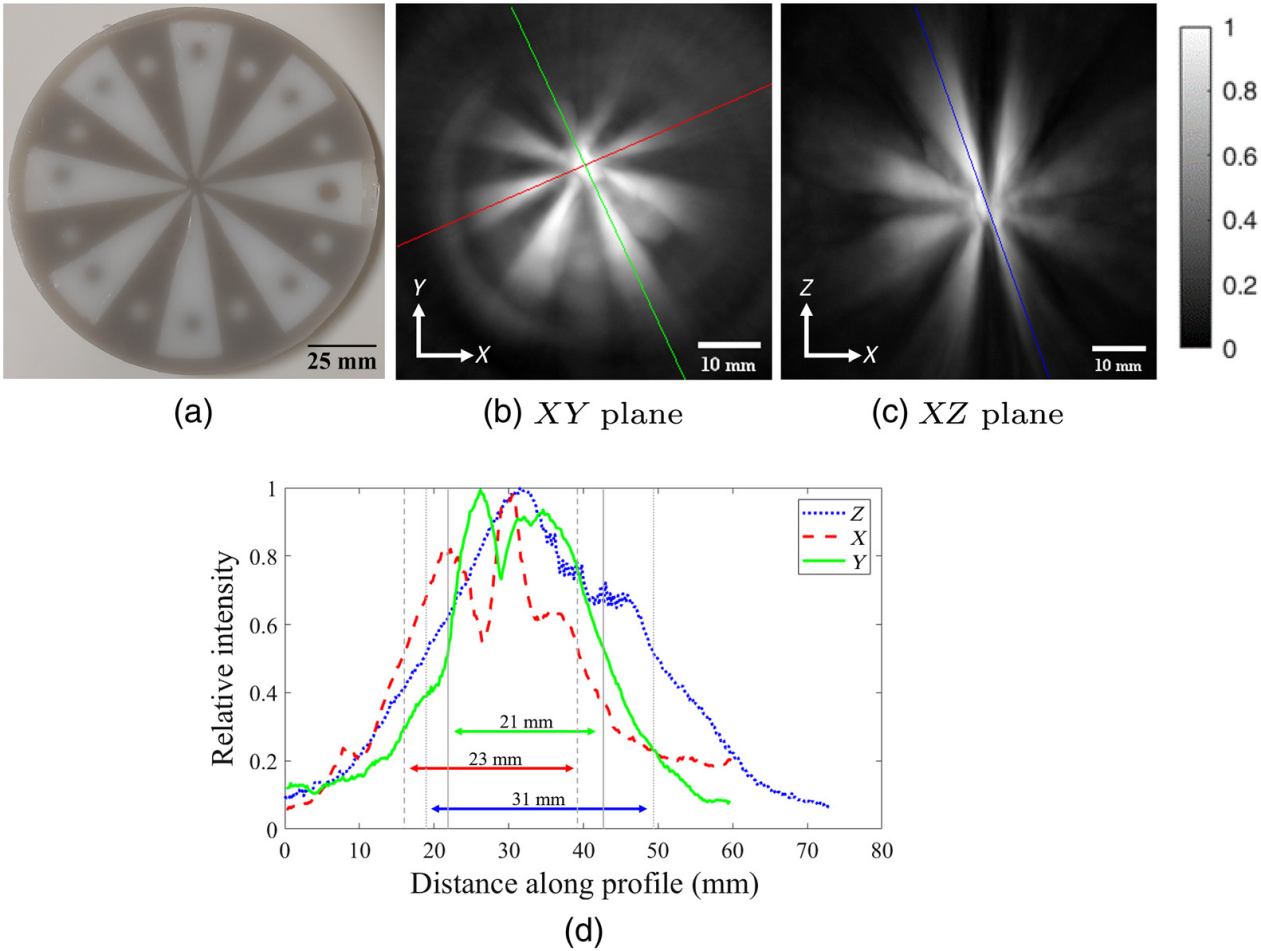
(a) Photo of the star phantom. PAT images from angular scans (single FoV) with the phantom oriented in the (b) horizontal XY plane and (c) vertical XZ plane. Note that the images shown are centered around the system’s axis of rotation, and the center of the phantom was slightly offset during imaging. (d) Example lines are shown with the corresponding line profiles and FWHM. PAT images (b) and (c) are grayscale and are scaled to maximize the full dynamic range within each reconstructed image. The intensity bar applies to both of these images.

To determine the FoV of a single angular scan, the central portion of the star phantom was imaged twice [Fig. 9(a)], first with the phantom lying horizontally in the XY plane [Fig. 9(b)], then rotated to lie vertically along the XZ plane [Fig. 9(c)]. For each cross-sectional image, line profiles were taken across the bright/absorbing star slices [Fig. 9(d)] and FWHM values were found to range between 21 and 31 mm isotropically. To ensure sufficient overlap and avoid any “hatch” patterns on images, imaging was subsequently completed with angular scans repeated at 15-mm intervals.

### 3.4 Star Phantom

**Fig. 10:**
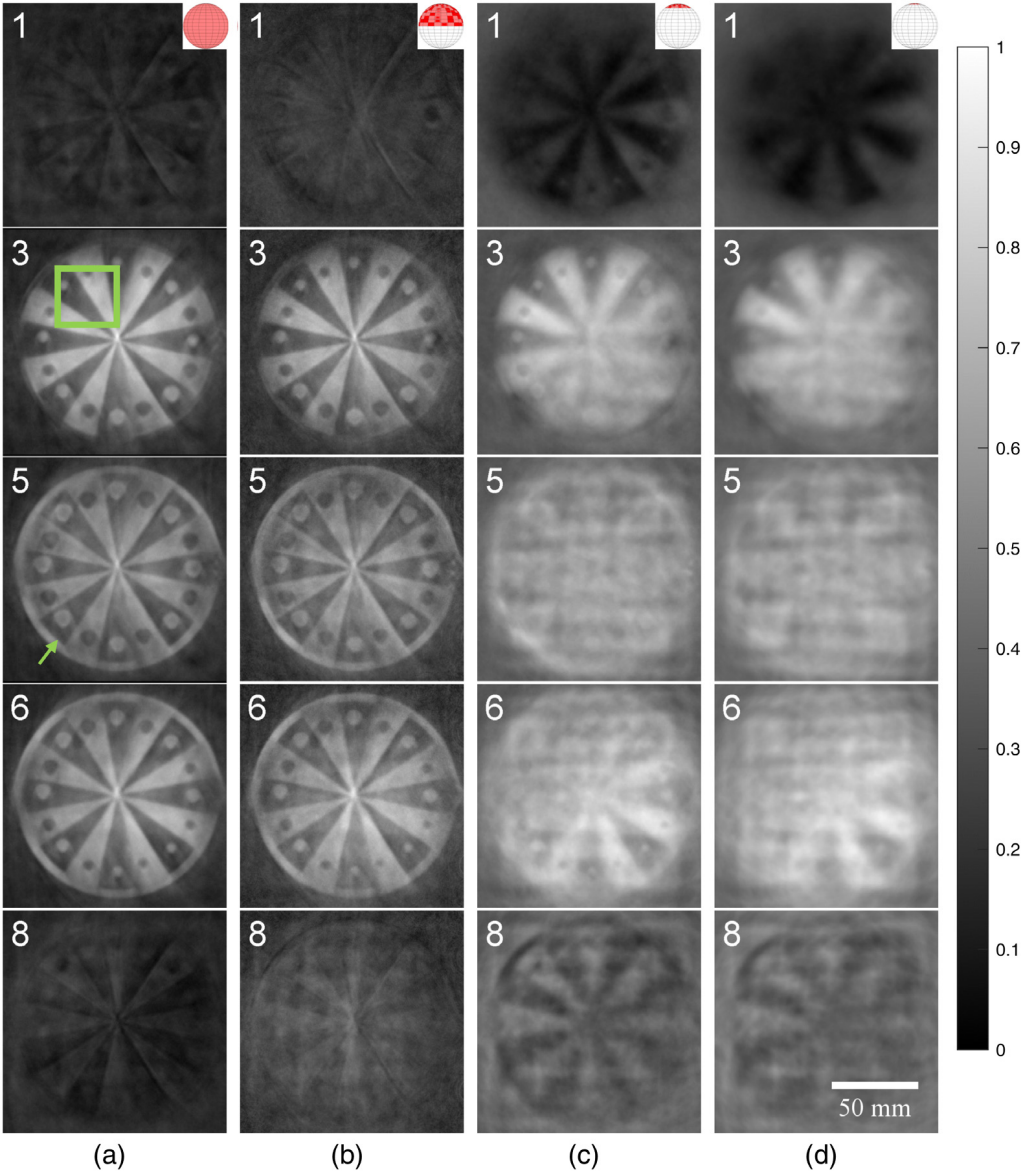
Reconstructed images of the star phantom for four different array geometries (columns) and five representative slices through the phantom (rows). Images using (a) the full ring array, (b) the top half of the ring array, (c) the top 16 detection elements on the ring, and (d) the top eight detection elements. Each slice is 0.25 mm thick, numbered in 2-mm steps, with 0.25-mm×0.25-mm pixel size, is grayscale and is scaled to maximize the full dynamic range within each montage. The green box represents where SNR values were calculated, while the green arrow points to an example of a streaking artifact. The intensity bar applies to all images.

The agarose phantom described in Sec. 2.2.3 was fully imaged by repeating the angular scan horizontally in a grid-like pattern with 15-mm steps at seven positions in X, and seven positions in Y, for a total of 49 repeated angular scans or 3626 total array positions. A montage of the reconstructed images is shown in Fig. 10 with representative 0.25-mm thick slices, with each slice number incrementing in 2-mm steps. Looking at Fig. 10(a), which uses the full array (near-full-view), the contrast between sections in each image can be clearly seen, with each embedded sphere also fully and well defined. The contrast and signal quality appear even across slices. All features of the phantom are clearly identifiable, albeit with slight intensity fluctuations in the middle slices (e.g., slice #5). In addition, there appear to be some streaking artifacts primarily near the spherical features. Moving to Fig. 10(b), which uses a hemispherical array with the imaged object just inside the boundaries of the hemisphere, the images are almost identical to those in Fig. 10(a). The contrast appears slightly lower and decreases as the slice number increments. Figures 10(c) and 10(d) show this to a much higher degree and also appear blurrier as slices increment, to the point that it is difficult to determine what the object is in the majority of the slices.

Quantitatively, using the area in the green box in slice #3, the full array image had an SNR of 2.42; the hemispherical array, 2.00; with the top 16 elements, 1.86; and with the top eight elements, 1.81.

### 3.5 Synthetic *Ex Vivo* Breast Tumor Phantom

**Fig. 11:**
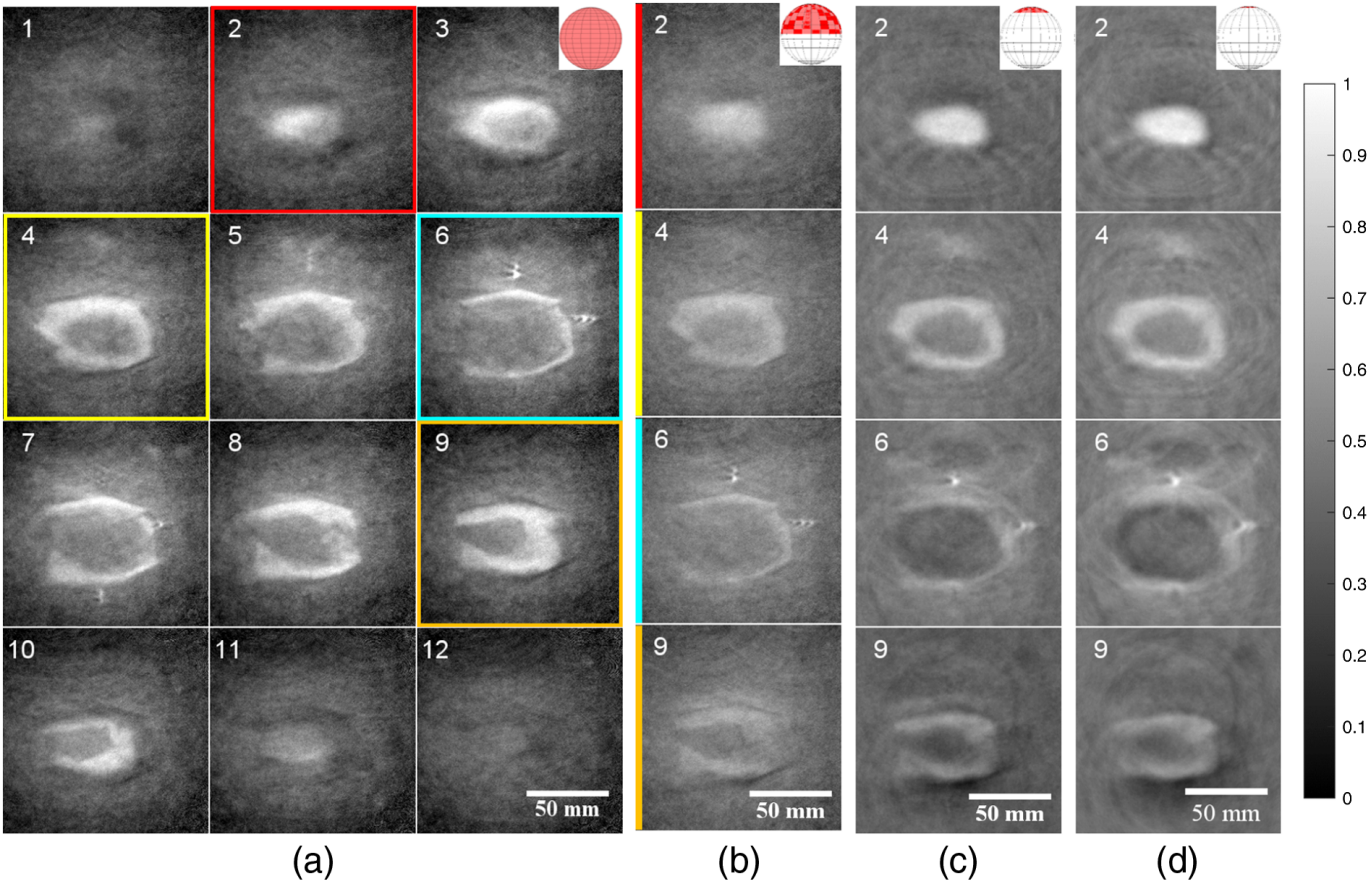
Tumor phantom made of chicken breast (core) and pork belly (outside). (a) Full-view PAT images of the phantom, from the top left to bottom right. Select images using (b) the top half of the ring array, (c) the top 16 detection elements on the ring, and (d) the top eight detection elements. PAT images are oriented to match Fig. 2(b). All images have 1.5-mm slice thickness, have 0.5-mm×0.5-mm pixel size, are grayscale, and are scaled to maximize the full dynamic range within each montage. Colored boxes in (a) designate slices shown in (b)–(d).

As described in Sec. 2.3.2, a tumor phantom made of chicken and pork fat was imaged. Referring to Fig. 2(a) as the top and Fig. 2(b) as the bottom, the full-view PAT image is shown as a montage of XY slices moving from the top to the bottom in 1.5-mm thick slices [Fig. 11(a)]. Note that the PAT 2D slices were oriented to match the bottom view [Fig. 2(b)] to better visually identify object features. The surface absorption was quite visible starting in slice 2, and moving through the slices, this remained the case at the edges of the specimen. Slices 3 to 10 showed a darker (negative contrast) core, which remained right up to the final bottom slice, matching up with the fat-free gap. Slices 2, 4, 6, and 9 from Fig. 11(a) were also reconstructed using subsets of the ring array as previously described [Figs. 11(b)–11(d)]. In the hemispherical reconstruction, overall contrast appeared lower than the full-view scenario, with particular emphasis on slices 6 and 9, where the edges were more difficult to make out. In slice 2, the contrast appeared to improve as the limited-view increased, as opposed to slices 4, 6, and 9, where the opposite was the case. Artifacts in the middle-top section of slices 4 and 6 worsened with increasing limited-view, with some streaking also appearing in slice 9.

### 3.6 Vascular Phantom

**Fig. 12:**
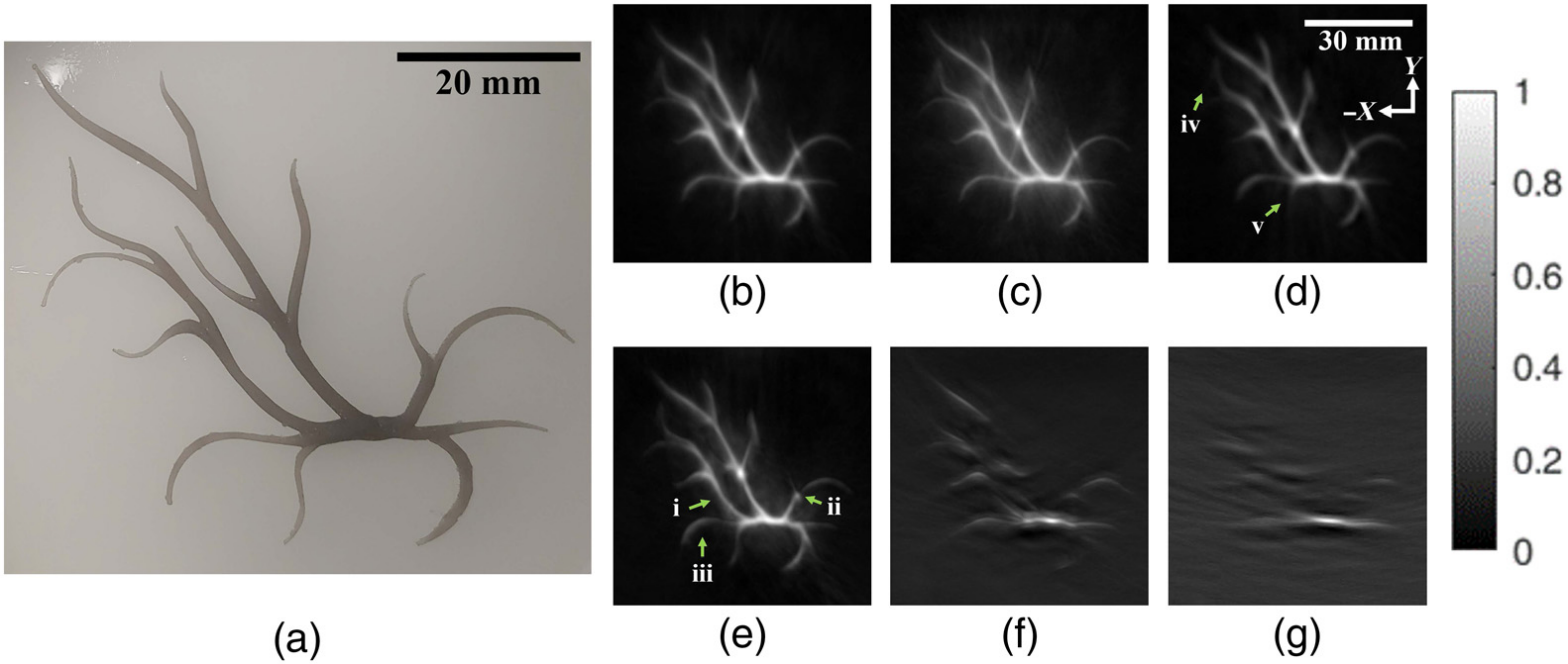
Vascular phantom 3D-printed, embedded in scattering agarose, and imaged with PAT. (a) Photo of the phantom prior to being embedded. Top-down MIP PAT images using (b) all the detectors, (c) high-frequency detectors, (d) low-frequency detectors, (e) a hemispherical (2π steradians) array of detectors located toward the positive Y direction, (f) ≈1.5π steradian coverage, and (g) ≈0.4π steradian coverage. All PAT images are grayscale and are scaled to maximize the full dynamic range within each reconstructed image. Green arrows denote regions of interest as compared to (b). (i) Narrower blood vessel. (ii) Artifact with missing signal due to limited-view angle. (iii) Smearing away from the detector array. (iv-v) Missing blood vessels. The intensity bar applies to (b)–(g).

The 3D-printed vascular phantom was imaged as described in Sec. 2.3.1. A photo of the phantom is shown in Fig. 12(a), prior to being embedded in optically scattering agarose. Figures 12(b)–12(g) are top-view MIP PAT images reconstructed using (b) all the detectors, (c) high-frequency detectors, (d) low-frequency detectors, (e) a hemispherical (2π steradians) array of detectors located toward the positive Y direction, (f) ≈1.5π steradian coverage, and (g) ≈0.4π steradian coverage.

Qualitatively, Fig. 12(b) showed the best contrast while still capturing the narrower vasculature. With only high-frequency detector elements [Fig. 12(c)], the edges of the vasculature were the sharpest, but had more background noise. Figure 12(d), with only low-frequency detector elements, appeared to be well differentiated from the background but struggled with displaying the narrowest vasculature [Fig. 12(d)(iv-v)]. Figure 12(e), with half the view-angle coverage, demonstrated smearing of the signals on the side away from the detectors [Fig. 12(e)(iii)] and had less consistent vessel thickness [Fig. 12(e)(i)]. Finally, images reconstructed using <2π steradian detector coverage [Figs. 12(f)–12(g)] displayed only the portions of the vasculature directly tangential to the axis of the detectors.

In addition, SNR measurements were taken for each MIP using a mask of the CAD model as reference. Figures 12(b)–12(e) were similar, with Fig. 12(b) having an SNR of 2.12 followed by Figs. 12(c)–12(e) with values of 2.02, 2.07, and 1.96, respectively. Figures 12(f)–12(g) had much lower SNR values at 1.83 and 1.39, respectively.

## 4 Discussion

We have developed a PAT system that incorporates a circular ring array and 6-axis robot to achieve a nearly closed, spherical, full-view detection geometry. Angular coverage for each FoV was ≈3.8π steradians. In the process, we also experimentally tested the use of negativity artifacts in the optimization of scanning PAT systems.^40^ By looking at maximum negative values in reconstructed images, we found that beyond $2^{12}$ effective detectors, image quality did not improve appreciably. The result corresponded with previously reported simulations.^40^ This system was then used to experimentally demonstrate the effects of limited-view angle on images of a simple sphere, a complex agarose star phantom, a synthetic breast tumor phantom, and a vascular phantom.

When exploring our results, it can be seen that in the negativity analysis (Fig. 6), each data series did not perfectly agree with each other, with different intensity negative values remaining in all images (Fig. 5). These were likely primarily due to (1) the glass pipette used to hold the absorbing sphere in place, (2) the small gap in detector coverage, and (3) oversampling of the signals at the top and bottom of the spherical detection surface. The glass pipette was used due to its stiffness, small size, and low optical absorption; however, it also had a significant impedance mismatch with the surrounding water. This would be expected to cause distortions in the acoustic signals and result in artifacts near the pipette location,^46^ compounded by the gap in detectors necessary to allow the pipette to extend into the imaging space. The artifacts are visible in the positive X direction in the left and center columns of Fig. 5.

With regards to spatial oversampling of the signals, as mentioned in Sec. 3.2, we had designed the array with a larger gap between modules at the top and bottom of the ring array to specifically reduce this effect. In addition, initial analysis revealed that omitting a further eight detector elements in those areas reduced negative values even further. Despite this, some negative signals remained in the images in the Z direction (Fig. 5, center and right columns), indicating that some uneven sampling remained. Future work could focus on improving this further through weighting the incoming signals depending on the density of detectors at the detector array surface.

Comparing reconstructions in Fig. 7, it becomes apparent why hemispherical detection arrays in literature are frequently considered sufficient. With simple objects, there were diminishing returns to pursuing full spherical coverage. However, image quality suffered as soon as the imaged object was outside of the region bounded by the hemispherical array as shown in Figs. 7(c) and 7(d). This was expected, as this was previously described analytically and in simulation,^10^ and tested in 2D.^47^ In addition, Fig. 8 shows how even when doubling the density of detectors in a hemispherical array to match the total number in the spherical array reconstruction, it cannot achieve the same image quality (as determined by negativity analysis).

When looking at the star phantom and synthetic breast tumor phantom, the advantages of a full-view spherical system were more apparent. In all the limited-view scenarios that we tested, there were gradual SNR reductions moving away from the detectors, which was absent in the full-view reconstructions. This effect was magnified when imaging thicker objects and as the limited-view scenario worsened. With decreasing view angle, the lateral resolution of the images also suffered and this again worsened moving away from the detectors. With the vascular phantom, we also saw smearing of the signals away from the detectors with the hemispherical setup [Fig. 12(c)(iii)]. When angular coverage was reduced even further, entire sections of the vasculature disappeared due to the anisotropic acoustic emissions being lost [Figs. 12(f)–12(g)]. Finally, the effects of spatial oversampling were exposed. As just discussed, even with a full-view angle, negativity artifacts appeared when signals were spatially oversampled in some direction(s) (Fig. 7). If we consider limited-view angle situations to be essentially extreme cases of spatial oversampling, then we can see how with an ideal spherical array, there are always transverse detectors to average out negativity artifacts. In addition, a full-view spherical array provides isotropic resolution as each dimension is in the axial plane for some detector.

Quantitatively, SNR comparisons agreed with the qualitative results. With the star phantom in Sec. 3.4, SNR values decreased going from full-vew to the most severely limited-view scenario. Similarly, SNR values with the vascular phantom (Sec. 3.6) followed the same trend. However, caution must be used when interpreting the results due to the small differences. While metrics such as SNR and contrast-to-noise have traditionally been used in imaging, their numerical values are not always relevant when the imaging target is easily distinguishable from the background as in this case.^48^

Both detector types used in this system were designed to complement one another in frequency response, which they do as shown in Fig. 12. However, when combined as in this study, the lowest common denominator in terms of pure spatial resolution appeared to fall on the low-frequency detectors and as such, the spatial resolution when using all the detectors was 2.0 mm, the same as with only low-frequency detectors. This may be because the two ranges of band-limited time series data were essentially overlaid, rather than being filtered and combined prior to reconstruction. In addition, while the detectors were considerably wideband, their covered range still did not extend high enough and as such, still contributed to negativity artifacts in the reconstructed images due to limited bandwidth even though angular coverage was nearly complete.^40^ In the future, we will consider reconstructing images with each detector type separately and overlaying the images, optimally weighting the signals from each detector type differently depending on the absorber geometry, and correcting for potential phase mismatches between the sensor types.

An idealized perfect detection array would require highly sensitive, yet omnidirectional detection elements. This would allow any imaged object within the sphere to be accurately imaged with a single acquisition, or in the case of our ring array, with a single angular scan. As this was not achievable with our PAT system since it had an FoV of ≈25  mm in diameter, we used a series of 15-mm Cartesian steps to image volumes up to ≈120  mm×120  mm×120  mm in dimension. While not ideal, this provided a good compromise between acquisition speed and image quality and is the approach taken by many other groups.^49^^–^^54^

Another area of interest in PAT is the speed of image acquisition. Image quality, detector sparsity, speed, and cost have constantly been at odds with each other but are also intertwined. Some recent literature have reported relatively rapid imaging, e.g., imaging an entire human breast in 15 s by scanning 512 detectors, but not all applications require such quick imaging.^2^ In this case, we were able to scan a ring array with one-eighth the number of detectors and adjust the sparsity of the resulting effective spherical array to the required application. While for this series of experiments our focus was not on achieving the fastest scanning speed, we still recorded a respectable ≈45  s per angular scan, which was then multiplied based on the size of the imaged object. The primary limitations in terms of decreasing scan time for this system were twofold. One, the use of a 10-Hz laser, and two, the slow transfer speeds from the DAQ boards to the computer. Combined, a faster, higher-power laser and a faster, modern DAQ could potentially reduce scan times by factors of 2 to 10× (depending on whether signal averaging was to be used).

Finally, it must be noted that most experiments in this paper used only a very simple delay and sum beamforming algorithm for image reconstruction, with little preprocessing of the signals nor postprocessing of the images. While there are many excellent reconstruction algorithms in literature, the focus of this work was to highlight the capabilities of this hardware geometry. Ongoing, we are exploring techniques such as directivity-weighting, coherence factor-weighting, and matched filtering as well as more recent reconstruction techniques to bring out the full potential of the system.

## 5 Conclusion

We have introduced a flexible, adaptable, scanning-type PAT system that can cover up to a 3.8π steradian view angle and therefore provide a near full-view closed spherical detection geometry. In literature, simulations comparing view angle coverage and geometries abound, but there are few experimental studies validating them. Moving forward, this system will also be used to explore and identify limited-view artifacts in clinical PAT imaging, specifically in situations such as tumor margin assessment and limb imaging.
